# *In Vitro* Evaluation of Some Endophytic *Bacillus* to Potentially Inhibit Grape and Grapevine Fungal Pathogens

**DOI:** 10.3390/plants12132553

**Published:** 2023-07-05

**Authors:** Oana-Alina Boiu-Sicuia, Radu Cristian Toma, Camelia Filofteia Diguță, Florentina Matei, Călina Petruța Cornea

**Affiliations:** 1 Faculty of Biotechnologies, University of Agronomic Sciences and Veterinary Medicine of Bucharest, 59, Mărăști Blvd., District 1, 011464 Bucharest, Romania; sicuia_oana@yahoo.com (O.-A.B.-S.); florentina.matei@biotehnologii.usamv.ro (F.M.); pccornea@yahoo.com (C.P.C.); 2 Research-Development Institute for Plant Protection, 8 Ion Ionescu de la Brad Blvd., District 1, 013813 Bucharest, Romania

**Keywords:** *Bacillus* spp., biocontrol, endophytes, grapevine, molds, trunk diseases

## Abstract

Romania has a long history of grapevine culturing and winemaking. However, like any agricultural sector, viticulture faces devastating biological threats. Fungi responsible for grapevine trunk diseases (GTDs) and grape spoilage lead to considerable yield losses and a decline in grapevine quality. In the actual context, many countries, including Romania, have reoriented their approaches to minimize chemical inputs, which have been proven to be toxic and to have negative impacts on the environment, and to replace them with sustainable biocontrol strategies for the wine-growing sector. Within biocontrol strategies, *Bacillus* spp. is a well-known plant-protective bacteria with antifungal properties. Within this paper, six endophytic bacteria from various plant sources were studied. The bacterial strains were identified as *B. pumilus*, *B. subtilis*, and *B. velezensis* by sequencing their 16S rDNA region. Regardless of the in vitro test methods (using living bacterial cells, bacterial-cell-free supernatant (CFS), and volatile active compounds (VOCs)), *B. velezensis* strains revealed strong and broad antifungal activity against grape and grapevine fungal pathogens such as *Aspergillus* spp., *Botrytis cinerea*, *Penicillium expansum*, *Diplodia seriata*, *Eutypa lata*, *Fusarium* spp., *Clonostachys rosea*, *Neofusicoccum parvum*, and *Stereum hirsutum*. The functional antifungal genes encoding for difficidin, fengycin, iturins, macrolactin, and mycosubtilin were molecularly detected, which could support the proven antifungal activity of the endophytic strains. Lytic enzymes involved in fungal growth inhibition, such as chitinase, cellulase, and proteases, were also revealed to be produced by some of these bacterial strains. Various other in vitro tests, such as phosphate and phytate solubilization, phytohormone synthesis, the production of enzymes involved in the polyamine biosynthetic pathway, and pH as well as temperature tolerance tests were carried out to reveal the plant-beneficial potential of these bacterial strains. These results revealed that the *B. velezensis* strains, especially BAHs1, are the most suitable endophytes for grapevine biologic control, which could lead to the future development of sustainable management strategies.

## 1. Introduction

Grapevine (*Vitis vinifera* L.) is one of the most cultivated crops worldwide due to the important economic value of wine, grapes, and dried grapes. Among EU states, Romania is among the first traditional wine-producing countries, with a vineyard area in 2021 that covered about 163,610 hectares with 1,005,280 tons of grape production and a wine production amount of 3.9 million hectoliters [1]. As with all other vineyards around the world, Romanian vineyards are confronted with different biotic and abiotic factors. Sometimes, stressful environmental conditions affect the quality and quantity of grapes and wine production, and can influence the vineyard’s safety [2,3,4,5,6,7]. Among the detrimental factors, the increasing incidence of fungal pathogens that severely affect different perennial organs of the grapevine has become a major concern for farmers and authorities [7,8,9,10,11,12,13,14]. However, most studies were focused on pathogens with immediate impacts on plant productivity, such as downy and powdery mildew [2,15,16], and spoilage fungi affecting grapes and wines, such as *Botrytis cinerea* (the causal agent of gray mold) [17,18,19], *Aspergillus* sp., mainly from section *Nigri* (involved in mycotoxin contamination) [5,13,20,21,22], and *Penicillium* spp. (producers of off-flavors) [5,13,18,20,22,23]. Currently, the major threat to the wine-growing sector is the incidence of GTDs, which induce a progressive decline in grapevines, and, consequently, important losses in wine production [7,14,24,25,26]. GTDs are caused by a group of fungal pathogens that affect the woody parts of the grapevine. Esca, Eutypa dieback, and Botryosphaeria dieback are three of the GTDs frequently detected in vineyards worldwide [7,24,25,26,27,28,29,30,31,32,33]. *E. lata* (the causal agents of Eutypa dieback), *Stereum hirsutum*, *Phaeoacremonium aleophilum*, *Phaeomoniella chlamydospora*, and *D. seriata* (the causative pathogens of Petri’s disease) are noted as being present in Romanian vineyards [11,12,14,34,35]. On a low basis, pathogenic fusaria were also isolated from Romanian vines affected by GTDs [36]. Recent studies have also reported pathogenic *Fusarium* species occurring in large numbers in Canadian nurseries and vineyards [37]. Pathogenicity studies have shown that some *Fusarium* species can cause necrosis in vines under stress conditions [37].

Unlike other fungal diseases, suppressing GTDs is more challenging as these pathogens are installed inside the wood and remain asymptomatic for a longer time [7,31,32,33,38]. These specific infections make it difficult for winegrowers to predict the risks and implement preventive control methods to minimize the development of spoilage fungi and GTDs [10,31,32,33,39]. Generally, fungal disease management is focused on applying combined control strategies using different prophylactic, cultural, biological, and chemical practices [31,38,40,41,42,43,44,45]. Most prophylactic and cultural measures can be applied preventively in nurseries and vineyards by removing the affected pruning residues, followed by the application of a fungicide on the resulting wounds either through spraying an appropriate formulation or brushing fungicides on the large pruning wounds and truck cracks [31,38,40,41,42,43,44,45,46,47]. Once GTDs are installed inside the wood and grapevine vascular tissue, phytosanitary treatments are less effective compared to other disease control measures [7,31,32,47]. The lack of efficient treatments and the difficulties in following disease management protocols maintain the interest of researchers in finding alternative plant protection measures to suppress GTDs. Moreover, regarding chemical treatments with benzimidazoles, dithiocarbamates, triazoles, strobilurines, and others, they are effective against only some of the pathogens involved in GTDs, and sometimes the application procedure is difficult to carry out on a wide scale in the vineyard [38]. Currently, one of the main objectives of the European Union is to reduce the chemicals used in agriculture in order to minimize their impact on the environment, and to consequently produce safer agricultural products for human consumption. Therefore, the transition from conventional to sustainable agriculture is one of the greatest challenges and the reason why biological plant protection products should replace some of the conventional synthetic formulations.

In recent years, treatments based on biological control agents (BCAs) have been reported as efficient alternatives to chemical protection methods, which are aligned with the context of safe and eco-friendly technologies that manage various plant diseases in nurseries and vineyards [15,21,48,49]. Most BCAs have the ability to colonize pruning wounds, protecting them from phytopathogenic infections. BCAs’ modes of action could involve nutrient and/or habitat competition, hyperparasitism, antibiosis via the biosynthesis of antimicrobial metabolites (microbial antagonistic compounds, enzymes, organic acids, or volatile compounds, etc.), and the induction of plant resistance [48,49,50,51,52,53,54,55,56,57]. Although numerous studies have been conducted for screening various biological agents, a few species of *Trichoderma* have been shown to have limited efficacy on GTDs, both in the nursery and in vineyards, as reviewed by Mondello et al. [38]. Currently, commercial *Trichoderma*-based products such as Esquive^®^ (*T. atroviride* I-1237), Vintec^®^ (*T. atroviride* SC1), and Remedier^®^, (*T. asperellum* ICC 012 and *T. gamsii* ICC 080) are available on the market to protect grapevines against different GTDs [58,59,60]. On the other hand, Bekris et al. [48] reported that the high incidence of beneficial endophytic bacteria could be involved in the significant reduction in GTDs. As many studies show, *Bacillus* is one of the most analyzed, appreciated, and powerful genera, as a reservoir of biocontrol agents [51,52,53,54,55,56,57]. In addition, *Bacillus* species are growing rapidly under minimal conditions, are spore-forming, and are able to produce a vast array of biologically-active compounds (such as a wide variety of extracellular enzymes, antibiotics, surfactants, and insecticidal toxins) [51,52,53,54,55,56,57]. *Bacillus subtilis*, *B. amyloliquefaciens*, and *B. velezensis*, isolated from a wide range of sources, have been reported as plant pathogen control agents due to their ability to biosynthesize numerous antifungal compounds, such as bacillaene, bacilysin, difficidin, fengycin, iturin, macrolactin, and surfactin [50,51,61,62]. Several studies also reported the effectiveness of *Bacillus* spp. in controlling spoilage fungi and GTDs [21,62,63,64,65,66,67]. A promising approach among BCAs is the use of beneficial strains with endophytic potential. Treatment with such colonizers will continuously protect the host plant after the intimate endophytic bond is established.

To our knowledge, this is the first complex study in Romania where *Bacillus* endophytes are proposed as biocontrol agents against grapevine fungal pathogens. The aim of this study was to evaluate, *in vitro*, the potential of endophytic *Bacillus* strains as promising biocontrol agents against a wide range of fungal pathogens responsible for GTDs and grape spoilage.

## 2. Results

### 2.1. Molecular Characterization of Bacterial Strains

#### 2.1.1. Bacterial Identification by Sequencing

The endophytic bacteria used in this study were identified at the species level based on their 16S rDNA sequence similarity to other bacterial strains from the NCBI database. The St 1T2 strain isolated from potato tubers was confirmed as *B. pumilus*. Its 16S DNA partial sequence revealed 100% identity and query cover with other 39 strains of *B. pumilus* from the NCBI database, including the ATCC 7061 strain (GenBank NR_043242.1). The sequences of *B. pumilus* St 1T2 strain can be found in the NCBI database under the accession GenBank no. OQ302768.

The bean root endophyte E1Pv was attributed to *B. subtilis*, with 99.93% identity and 100% query cover with the other 68 strains from the NCBI database. The 16S rDNA sequence of this strain is available on NCBI under GenBank no. OQ305856.

Among the studied bacteria, all three seed endophytes (BAHs1, BPVs2, BTAs3), as well as the leaf and flowers endophyte from lavender (LFF MYM 5), were attributed to *B. velezensis*. Their sequence can be downloaded from NCBI using the following accession numbers: OQ305996 (for BAHs1 strain), OL762398 (for BPVs2 strain), OQ305956 (for BTAs3 strain), and OQ304006 (for LFF MYM 5 strain). Moreover, these *B. velezensis* strains share a 1392 bp identical sequence within their 16S rDNA gene, as can be revealed through partial sequencing.

**Figure 1:**
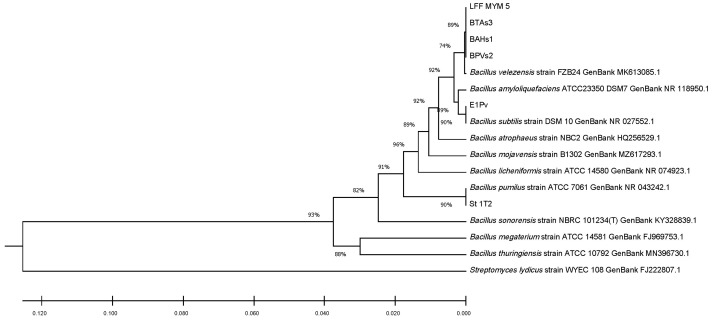
Phylogenetic tree constructed with MEGA X based on the 16S rDNA sequences using ClustalW alignment tool and UPGMA clustering analysis.

The 16S ribosomal DNA sequences of the studied endophytic strains were compared to those of seven reference strains from the same species, using MEGA X software and 16S rDNA sequences of reference strains downloaded from the NCBI database. The alignment was made in ClustalW, while the phylogenetic tree was built using the UPGMA clustering analysis (Figure 1). The phylogenetic tree obtained complied with most of our identifications at the species level.

#### 2.1.2. PCR Detection of Functional Genes

**Figure 2:**
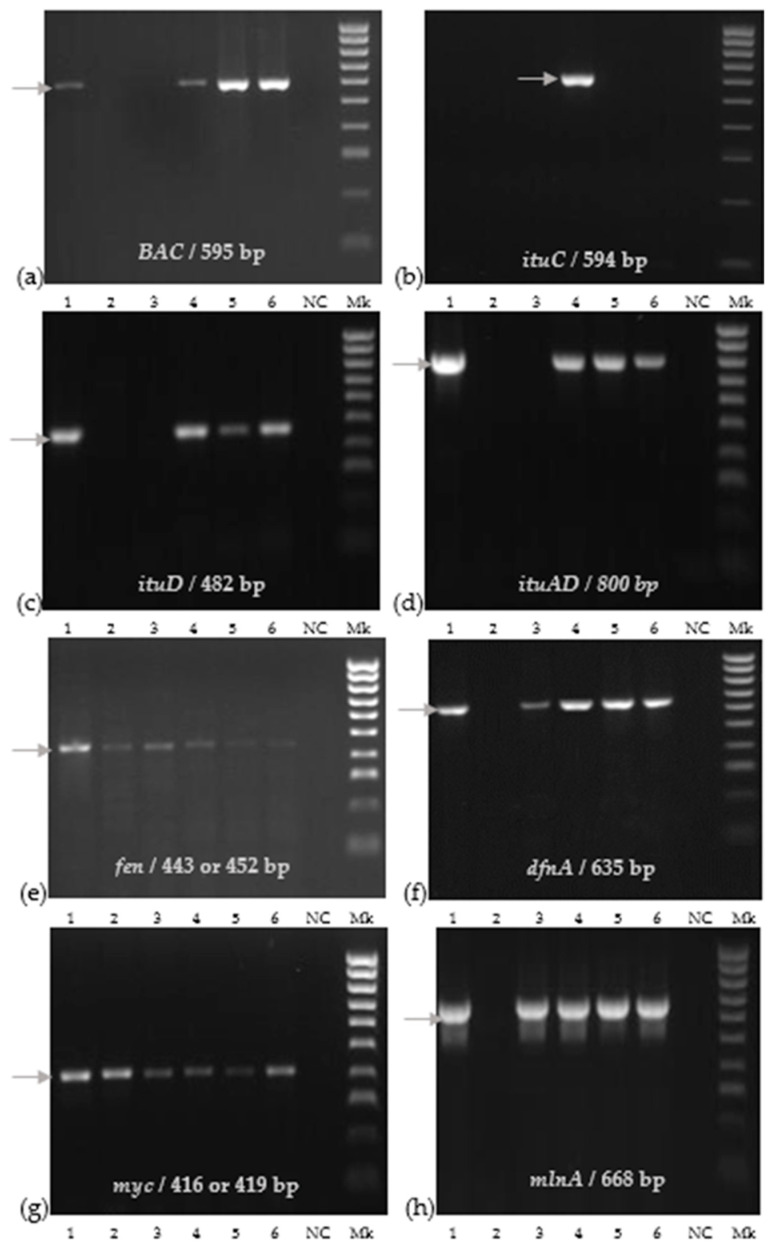
Molecular detection of functional genes of bacterial strains: (**a**) *BAC*; (**b**) *ituC*; (**c**) *ituD*; (**d**) *ituAD*; (**e**) *fen*; (**f**) *dfnA*; (**g**) *myc*; (**h**) *mlnA.* The corresponding lanes are as follows: 1: LFF MYM 5; 2: St 1T2; 3: E1Pv; 4: BPVs2; 5: BAHs1; 6: BTAs3; NC: negative control; Mk 100 bpDNA ladder.

We detected eight genes through PCR, encoding for bacillibactin, difficidin, fengycin, iturins, macrolactin, and mycosubtilin (Figure 2).

The St 1T2 strain identified as *B. pumilus* showed positive bands only for *fen* and *myc* genes, and the E1Pv strain identified as *B. subtilis* had a positive amplification of *fen*, *dfnA*, *myc*, and *mlnA* genes, respectively (Figure 2). *Bacillus velezensis* BPVs2, BAHs1, BTAs3, and LFF MYM 5 strains expressed PCR products corresponding to seven functional genes including *BAC*, *itu D*, *itu AD*, *fen*, *dfnA*, *myc*, and *mlnA* genes. The positive detection of the *ituC* gene was only observed for the *B. velenzensis* BPVs2 strain (Figure 2).

Additionally, a PCR analysis revealed the presence of all studied functional genes related to the synthesis of antifungal compounds in *B. velezensis* BPVs2.

### 2.2. Antifungal Efficiency of Bacterial Endophytes

**Figure 3:**
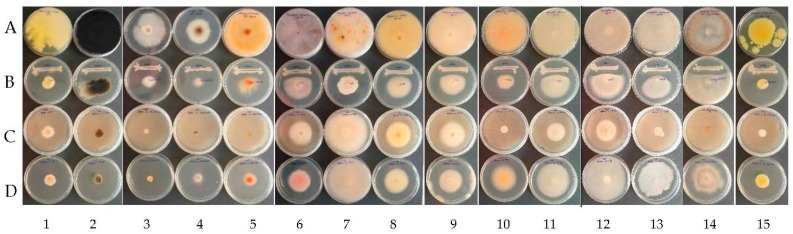
Examples of antagonistic activity of *B. velezensis* BAHs1 A—control (fungal strains); B—dual antagonism assays; C—bacterial VOCs; D—bacterial CFSs against grapevine trunk diseases and grape spoilage fungi (left to right) 1: *C. rosea* MI1; 2: *N. parvum* MI 25; 3: *D. seriata* CBS 151.24; 4: *E. lata* CBS 208.87; 5: *S. hirsutum* CBS416.61; 6: *F. oxysporum* MI 3; 7: *F. equiseti* MI 6; 8: *F. solani* MI 13; 9: *F. proliferatum* MI 4; 10: *A. ochraceus* MI 2; 11: *A. flavus* MI 24; 12: *A. niger* MI 5; 13: *A. carbonarius* MI 15; 14: *Botrytis cinerea* MI Aligote Husi; 15: *P. expansum* MI BB Husi.

The spectrum of antifungal activity was investigated in vitro against 15 pathogenic fungi associated with grapevine trunk diseases and grape spoilage through a dual culture plate assay, agar diffusion assay of the CFSs, and an inverted Petri dish assay for VOCs (Figure 3).

#### 2.2.1. *In Vitro* Screening of the Antifungal Activity of Bacterial Strains

The antifungal activity of bacterial endophytic strains was emphasized, *in vitro*, through a dual culture plate assay. Our results showed that the *B. velezensis* strains significantly suppressed the mycelial growth of all pathogenic fungi (ranging from 40.0% to 87.4%) compared to the control (Table 1). Among all strains, BAHs1 seems to be the most effective in inhibiting fungal growth. *B. subtilis* significantly inhibited the growth of pathogenic fungi (except for *A. flavus*), but less than *B. velezensis*. The *B. pumilus* St 1T2 strain displayed highly varying degrees of fungal growth inhibition, ranging from 0.0 to 62.9%.

**Figure 4:**
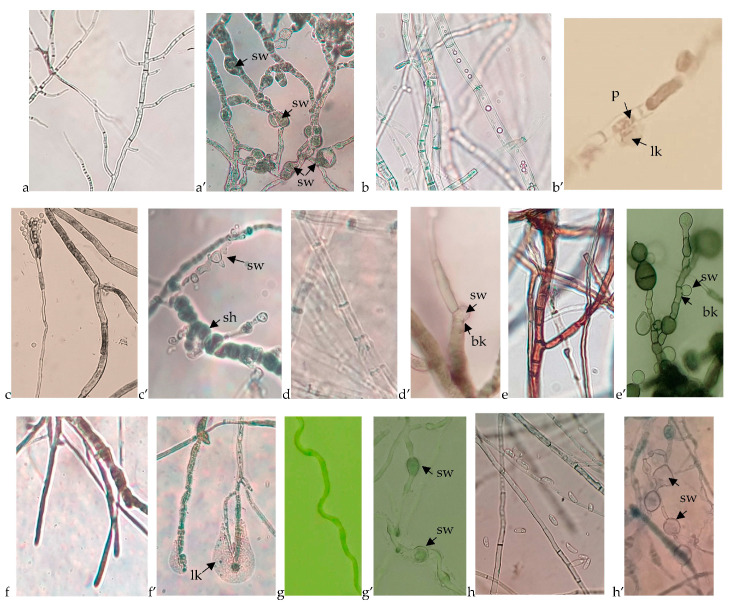
Microscopic view of the untreated fungal growth compared to the fungal disruption caused by the *B. velezensis* BAHs1. (**a**) *A. carbonarius*, (**a’**) swelled cells (sw) of *A. carbonarius*; (**b**) *Botrytis cinerea*, (**b’**) cell perforations (p) and cytoplasm leaking (lk) of *Botrytis cinerea*; (**c**) *P. expansum*, (**c’**) shortened (sh) and swollen cells (sw) of *P. expansum*; (**d**) *D. seriata*, (**d’**) cell wall breaking (bk) and swollen cytoplasmatic membrane of *D. seriata*; (**e**) *N. parvum*, (**e’**) cell wall breaking (bk) and swollen cytoplasmatic membrane of *N. parvum*; (**f**) *S. hirsutum*, (**f’**) cell wall and membrane degradation as well as cytoplasm leaking of *S. hirsutum*; (**g**) *E. lata*, (**g’**) swelled cells of *E. lata*; (**h**) *F. oxysporum*, (**h’**) swelled cells of *F. oxysporum*.

Results related to fungal growth disruption caused by the *B. velezensis* BAHs1 were compared to the growth morphology of the fungal controls. A microscopic analysis of bacterial–fungal interactions revealed some mycelia growth disruptions, hyphal swelling or cell lysis, and the cytoplasmic leaking of various pathogens due to bacterial antifungal activity (Figure 4).

#### 2.2.2. Evaluation of the Antifungal Activity of CFSs

Bacterial CFS was also assessed for antifungal properties using the qualitative agar diffusion method (Table 2). Among the *Aspergillus* sp., *A. ochraceus and A. carbonarius* were the most sensitive fungi to CFSs fermented by *B. velezensis* strains. Moreover, the CFSs derived from *B. velezensis* strains exhibited more than 50% inhibition in most tested pathogenic fungi associated with GTDs, except for *E. lata* which was inhibited, ranging from 39.5% to 49.3%. Also, these CFSs had a lower inhibitory effect, ranging from 20.7% to 41.6%, against almost all *Fusarium* sp. tested, except for *F. equiseti*, which was the least inhibited (4.0–7.2%) (Table 2).

Overall, the CFSs of *B. subtilis* E1Pv and *B. pumilus* St 1T2 strains displayed a lower inhibitory effect (up to 20%) on the mycelium growth of the tested fungi, except for *D. seriata* (inhibition ratio 44.5% and 27.8%), *P. expansum* (46.4% and 43.6%), *N. parvum* (59.8% and 46.4%), and *C. rosea* (60.1% and 54.7%) (Table 2).

#### 2.2.3. Evaluation of Antifungal Activity of Bacterial Endophytes VOCs

All selected endophytic bacteria produced VOCs. The VOCs secreted by *B. velezensis* strains determined a strong inhibition of mycelial growth of *A. carbonarius* by 67.4–76.3%, *A. ochraceus* by 62.9–73.8%, *A. flavus* by 42.8–49.1%, and a lower inhibitory effect of *A. niger* by 10.5–18.2% (Table 3). Moreover, these VOCs strongly inhibited the growth of more than 69.0% of *P. expansum* and more than 77.9% of *Botrytis cinerea*.

The VOCs derived from *B. subtilis* E1Pv and *B. pumilus* St 1T2 produced a significantly delayed growth of *A. carbonarius*, *A. ochraceus*, and *A. flavus* and had the lowest inhibitory effect on *A. niger* (Table 3). On the other hand, both VOCs exhibited a higher inhibitory effect against *Botrytis cinerea* and *P. expansum.*

In regard to trunk fungi, VOCs secreted by all bacterial strains strongly suppressed the mycelium growth of *S. hirsutum* by 74.0–85.8%, *N. parvum* by 65.5–77.4%, *E. lata* by 59.4–78.2%, *D. seriata* by 54.5–83.7%, and *C. rosea* by 39.2–55.8% (Table 3). Moreover, all VOCs were more efficient against *F. proliferatum* (inhibition rate of 32.9–43.8%) and *F. oxysporum* (inhibition rate of 16.3–20.5%) than against *F. solani* and *F. equiseti* (Table 3).

### 2.3. Enzymatic Profiles of Bacterial Strains

In order to obtain an overview of the enzyme profile, the bacterial strains were tested both on the culture media supplemented with different substrates, as well as with the API ZYM test (Table 4 and Table 5).

Alfa-amylase production was revealed by all strains, except St 1T2, which was isolated from a healthy potato tuber. This aspect can be explained, as the St 1T2 strain did not affect the integrity of its host’s tubers by hydrolyzing plants’ storage carbohydrates. All strains, except BPVs2, were positive for ACC-deaminase. All tested strains revealed positive reactions for arginine-decarboxylase and ornithine-decarboxylase, based on the pink color developed on specific media. All tested strains revealed cellulase activity. None of the endophytic strains revealed xylanase activity. Chitinase activity was evaluated due to its role in plant protection. However, only the E1Pv revealed chitinolytic activity, with a weak degradative potential. Lipolytic activity was registered with St 1T2 and E1Pv.

According to the API ZYM system, all tested strains exhibited esterase (C4) and esterase lipase (C8) activities (Table 5). Except for the St 1T2 strain, all tested strains presented alkaline phosphatase. The α- and β-glucosidase activity was observed in the E1Pv strain, and α-chymotrypsin activity was observed only in the St 1T2 strain. *B. velenzensis* LFF MYM 5 and BAHs1 strains were positive for naphthol-AS-BI-phosphohydrolase. *Bacillus velenzensis* strains showed a positive reaction to acid phosphatase, except for the LFF MYM 5 strain.

### 2.4. Biochemical Characterization of Bacterial Strains

All *Bacillus* strains had the ability to produce ammonia (Table 6). Except for the BPVs2 strain, all tested strains revealed a positive reaction to acetoin production. However, only St 1T2 and E1Pv strains showed a visible phosphate solubilizing halo. *B. velezensis* BAHs1 and LFF MYM 5 strains were able to produce surfactants, as shown by the clear halo zone.

The endophytic bacteria were also investigated for their ability to produce indole-3-acetic acid (IAA), in the presence or the absence of tryptophan, as a precursor. *B. velezensis* BPVs2, BTAs3, and LFF MYM 5 strains showed a high IAA production in the tryptophan-free medium at 72 h. Our results showed that L-tryptophan stimulated the IAA production of all endophytic bacteria, except the BPVs2 strain. The highest IAA concentration was recorded by the E1Pv strain (8.62 ± 1.1 μg/mL), and the LFF MYM 5 strain (7.03 ± 1.1 μg/mL) after 72 h incubation. Thus, the duration of 72 h was selected. Except for the BPVs2 strain, the IAA biosynthesis carried out by bacterial endophytes was significantly induced by tryptophan by 1.3- to 3.6-fold compared with the same strains incubated in the medium not supplemented with the precursor.

### 2.5. Tolerance of Bacterial Strains at Different Abiotic Stress Factors

The tested pH parameters did not influence bacterial growth (Table 7). All strains were able to develop abundant growth after one day of incubation at 30 °C.

Some differences in growth were seen when bacteria were incubated at different temperatures (Table 7). At 10 °C, only the St 1T2 strain was able to develop colonies, with visible growth starting from the third day of incubation. The other bacterial strains remained latent at 10 °C. At 20 °C, all strains developed colonies after two days of incubation, while at 30 °C the colonies were developed overnight. At 40 °C and 50 °C, bacteria were able to develop colonies after less than 8 h incubation time.

Regarding copper tolerance, only *B. pumilus* St 1T2 exhibited a high tolerance to CuSO_4_ up to 700 mg/L. The *B. subtilis* E1Pv strain did not grow at 200 mg/L CuSO_4_ and higher concentrations, while the *B. velezensis* BPVs2 strain grew at CuSO_4_ concentrations up to 50 mg/L (Table 7).

## 3. Discussions

The role of *Bacillus* spp. as a plant growth-promoting bacteria (PGPB) has been demonstrated by numerous studies reported in the literature [51,52,53,54,55,56,57]. In our study, based on the 16S rDNA sequence analysis, the bacterial strains were identified as *B. velezensis* (four strains), *B. pumilus* (one strain), and *B. subtilis* (one strain). However, *B. velezensis*, *B. pumilus*, and *B. subtilis* species were all included in the *B. subtilis* species complex [68]. Such a taxonomic conversion is, nowadays, considered economically important for biocontrol strains used in plant protection products, such as FZB 42 (DSM2311), which was registered as a *B. amyloliquefaciens* strain, and is now reclassified as *B. velezensis* based on molecular genetic findings [69]. However, to confirm the taxonomic affiliation of our strains, a further molecular analysis should be performed. A suitable way would be to sequence other conserved regions of the bacterial chromosome, such as the *gyrA* and *gyrB* genes of the DNA gyrase subunits [70,71].

*Bacillus* spp. exhibit mechanisms (including a wide variety of extracellular enzymes, antibiotic lipopeptides, surfactants, hormones, etc.) against pathogenic fungi, making them useful for suppressing or reducing disease incidence [50,51,61,62]. Various key genes, which are responsible for the biosynthesis of lipopeptides, have been detected in the tested endophytic bacterial strains. These genes are responsible for the synthesis of cyclic lipopeptides, such as surfactin, bacillomycin, fengycin, and iturins, which suppress a wide spectrum of phytopathogenic fungi. This finding supports the antifungal activity of the bacterial strains presented in this study. In another study, the molecular detection of the genes involved in the biosynthesis of antifungal compounds such as bacillomycin (*bmyA*), iturin A (*ituA*), iturin D (*ituD*), fengycin (*fen*), and surfactin (*srf/lch*), from halotolerant *B. amyloliquefaciens* 24.5, was correlated with an excellent inhibitory activity against *Alternaria alternata*, *Aspergillus carbonarius*, *A. flavus*, *A. niger*, *Botrytis cinerea*, *F. oxysporum*, and *P. digitatum*, except for *Rhizopus* sp. [62].

The genus *Bacillus*, mainly *B. amyloliquefaciens*, *B. subtilis*, *B. siamensis*, and *B. velezensis*, are well-known biocontrol agents that suppress phytopathogenic fungi from various environments. Our experiments revealed that *B. velezensis* strains exhibited a wide and strong broad inhibition spectrum, both when they were tested in direct, *in vitro*, confrontations as well as indirectly through the use of their VOCs and CFSs against 15 pathogenic fungal species. Significant differences between the three tested antagonism methods were noticed for each of the tested bacterial strains (*p* < 0.05). Using living bacterial cells, the antifungal efficacy is higher compared to VOCs or CFSs alone (*p* < 0.05). This could be explained by the synergistic antifungal effect of VOCs and antifungal compounds released by the bacteria in the CFSs. This finding is supported by various studies conducted to assess the performance of *Bacillus* in controlling fungal diseases. In another in vitro evaluation, Diguta et al. [21] reported two epiphytic bacterial strains with strong inhibitory activity against grape spoilage fungi such as *A. carbonarius*, *Botrytis cinerea*, *F. oxysporum*, and *P. expansum.* The *Bacillus subtilis* PTA-271 strain, alone or in combination with *T. atroviride* SC1, has shown promising results for the control of the aggressive *N. parvum* Bt67, responsible for the Botryosphaeria dieback disease [60,65]. Different studies have described the biocontrol potential of *B. velezensis*, not only in vitro but also in protecting grapevine against gray mold disease [15], and table grapes against bunch rot caused by *Botrytis cinerea* post-harvest [66]. More recently, Bustamante et al. [67] showed that *B. velezensis*, *Pseudomonas chlororaphis*, and *Serratia plymuthica* strains, isolated from GTD-symptomatic and asymptomatic grapevine, had different mycelial inhibition degrees via direct and/or indirect mechanisms against *Diaporthe ampelina*, *Diplodia seriata*, *Lasiodiplodia theobromae*, *N. parvum*, *E. lata*, *Fomitiporia polymorpha*, *Ilyonectria liriodendri*, and *Phaeoacremonium minimum.* Various *B. velezensis* and *B. amyloliquefaciens* strains have been reported as well-known biocontrol agents against bacterial, fungal, and viral plant pathogens such as *Xanthomonas citri* subsp. citri [72], *Armillaria solidipes* [73], *Erysiphe heraclei* [74], *Phytophthora infestans* [75], *F. oxysporum* f.sp. *fragariae* [76], *F. oxysporum* f.sp. *cubense* [77], *P. digitatum* and *P. italicum* [78], *Sclerotinia sclerotiorum* [77], tomato spotted wilt virus, groundnut bud necrosis virus, and tobacco streak virus [77].

Differences have been noticed in regard to the intensity and mode of fungal growth inhibition from one phytopathogen to another. Microscopic analyses of the clear inhibition zones revealed some abnormal changes in the fungal growth architecture. However, the general trend of mycelial alteration caused by the biocontrol strains was similar toward the same fungi [79]. Differences among tested strains are hard to evaluate in terms of the intensity in mycelial alterations, but the generally tested *B. velezensis* strains were more active. Studies reporting similar mycelia growth disruptions are suggesting that the antifungal metabolites [80] and VOCs [81] released by biocontrol strains are involved in such hyphal alterations.

Our results revealed that the tested bacterial strains exhibited a wide spectrum of hydrolytic enzymes with potential in plant protection and growth promotion. In some cases, α-amylase production by plant growth-promoting bacteria is a beneficial trait, as it can be correlated with gibberellin phytohormone synthesis [82]. In plants other than grapevine, bacterial α-amylase release is also relevant in stimulating the seed germination process, triggering improved seedlings’ root and shoot growth [83]. The ACC-deaminase prevents increased ethylene levels in plants affected by biotic and abiotic stress conditions, and it also prevents premature senescence and plant cell death. ACC-deaminase-producing bacteria can reduce the deleterious effects caused by drought [84], various heavy metals [85,86], and salt stress [87]. Chitinolytic and cellulolytic enzyme production is described as a beneficial trait for plant protection [88,89]. The cellulase activity can also improve nutrient availability for plant growth-promoting bacteria [90]. This is considered a good trait among endophytes, as in the case of plant-associated microorganisms. However, the xylanase activity is correlated with the pathogenicity potential and virulence factor [91]. Xylanolytic enzymes induce necrotic lesions in the xylem vessels, disturbing water and nutrient transport within the plant. Some lipolytic enzymes are suggested to be involved in extracellular virulence factors [92], as well as in the stimulation of the plant defense response against fungal pathogenic attack, or the increases in the phytoalexin levels in plants for better protection against various stress factors [93].

Arginine-decarboxylase and ornithine-decarboxylase enzymes are able to initiate polyamine biosynthetic pathways. These polyamines are involved in many important cellular processes [94]. Moreover, they can represent the plant’s biostimulating activity against abiotic stress, especially in cold weather. For grapevine, this could be translated into plant protection against early autumn frosts which affect the immature wooded vine shoots, as well as late spring frosts, which affect the viability of young and immature tissues exposed to frost damage.

The tested bacterial strains were also characterized for their capacity to produce surfactants, acetoin, ammonia, solubilize phosphate, as well as indole acetic acid. All these characteristics are important when analyzing the potential of these *Bacillus* isolates to promote plant growth. Bahadir et al. [95] identified, among 440 *Bacillus* isolates from different sources, six phosphate solubilizers with a good amount of indole acetic acid, potentially qualifying them as promising biofertilizers. Rolli et al. [96] selected 15 bacterial strains isolated from grapevine roots with multiple beneficial traits, such as ACC deaminase activity, phosphate solubilization, indole-3-acetic acid production, siderophore production, ammonia production, and potential nitrogen fixation, to promote the growth of grapevines in the field.

The survivability of the *Bacillus* spp. to abiotic factors, such as temperature and pH variations, as well as copper toxicity, will allow their effective deployment to reach the desired beneficial effects. All bacterial strains displayed good tolerance over a wide pH range (5–11). In addition, all bacterial strains tolerated high temperatures of up to 50 °C, even though they remained latent at low temperatures (10 °C), except for the St 1T2 strain identified as *B. pumilus*. In viticulture, copper (Cu) compounds are the traditional and active ingredients used to combat the downy mildew (caused by *Plasmopara viticola*) and are used in some applications to manage the GTDs complex [97,98,99]. In our study, the *B. velezensis* BPVs2 strain tolerated the presence of low concentrations of copper (50 mg/L) and *B. subtilis* E1Pv (100 mg/L). Only the *B. pumilus* strain proved to be resistant to high concentrations of copper ions (up to 700 mg/L). The biocontrol efficacy of *B. velezensis* SZMC 6161J proved to be sensitive to elevated levels of metals [100].

## 4. Materials and Methods

### 4.1. Microorganisms and Growth Conditions

Six strains of bacterial endophytes isolated from different plant organs and species were used in this study. The LFF MYM 5 strain were previously isolated from lavender [79]. The St 1T2 strain was isolated as an endophyte from potato tuber, E1Pv strain from bean roots, while BPVs2, BAHs1, and BTAs3 strains were isolated from the seeds of beans, peanuts, and wheat kernels, respectively. In previous studies, these endophytic bacteria have been identified based on the Biolog technique as belonging to *Bacillus subtilis* group [79,101].

These bacteria were grown on Luria Bertani (LB, Carl Roth GmbH + Co.KG) at 28 °C. Broth cultures were incubated under orbital shaking at 150 rpm. Stock cultures were long-term preserved in LB Broth with 25% glycerol at −20 °C. Otherwise, cultures were maintained on solid media at 4 °C.

Fifteen fungal pathogens were used in this study as the reference phytopathogenic fungi for the antifungal test (Table 8).

These fungi were preserved as mycelial plugs in mineral oil (MP Biomedicals, Eschwege, Germany) at 4 °C. Cultures were routinely grown on potato dextrose agar (PDA, Merck™, Darmstadt, Germany) at 25 °C.

### 4.2. Molecular Analysis of Bacterial Strains

#### 4.2.1. Bacterial Genomic DNA Extraction

Fresh bacterial cultures, after 18 h in LB broth, were used for genomic DNA extraction. Mechanical cell lysis was performed using bead beating for 1 min with the Mini Bead Beat-er-8 homogenizer (BioSpec, Bartlesville, OK, USA) in order to improve DNA extraction. The bacterial DNA was then extracted using the High Pure PCR Template Preparation kit (Roche Life Science, Mannheim, Germany). DNA quantification was performed with the SpectraMax^®^ QuickDrop™ Micro-Volume Spectrophotometer (Molecular Devices, San Jose, CA, USA).

#### 4.2.2. Bacterial Strain Identification by Sequencing

The endophytic bacterial strains were identified at the species level based on the highly conserved 16S rDNA region. For this scope, the bacterial universal primers 27F (5′-AGA GTT TGA TCC TGG CTC AG-3′) and 1492R (5′-ACG GCT ACC TTG TTA CGA CTT-3′) were used to amplify the 16S rDNA sequence [102]. The PCR was performed in a 25 µL reaction volume with 20 ng of template DNA. The PCR mix contained 1X Buffer, 2 mM MgCl_2_, 0.2 mM dNTPs (Thermo-Scientific, Baltics, UAB, Vilnius, Lithuania), 0.5 μM of each primer, and 0.25 U of MangoTaq DNA Polymerase (BioLine, London, UK), all mixed in MilliQ water. Furthermore, the annealing temperature was set at 55.5 °C. The PCR products were revealed through agarose gel electrophoresis. The PCR products were then purified and sequenced through the Sanger dideoxy sequencing method, by CeMIA (Cellular and Molecular Immunological Applications, Greece). The partial sequences obtained with forward and reverse primers were aligned using the BioEdit Sequence Alignment Editor program, version number 7.2.5 (https://www.labtools.us/bioedit-sequence-alignment-editor/, accessed on 2 March 2022). The assembled sequences were further subjected to online NBLAST (Nucleotide Basic Local Alignment Search Tool) software (http://www.ncbi.nlm.nih.gov/BLAST/, accessed on 15 April 2022) available from the National Center for Biotechnology Information (NCBI) for taxonomic identification based on the sequence’s similarity with other microorganisms found in the NCBI database.

The phylogenetic analysis was performed using MEGA X software [103], version 10.1.8., and the 16S rDNA sequence’s alignment was made with ClustalW, while the phylogenetic tree was built using the UPGMA clustering analysis [104].

#### 4.2.3. Antifungal Functional Gene Identifications

Target genes encoding antifungal compounds (such as bacillibactin, difficidin, fengycin, iturins, macrolactin, and mycosubtilin) were detected via PCR using specific primers (Table 9).

The amplification program involved initial denaturation for 4 min at 94 °C, followed by 30 cycles of 30 s of denaturation at 94 °C, 30 s of annealing at optimum temperature (Table 9), and 75 s of elongation at 72 °C, with a final elongation for 7 min at 72 °C. All PCR products were revealed following 1.2% agarose gel electrophoresis in 0.5X Tris-Borate-EDTA buffer, supplemented with ethidium bromide. The DNA ladder of 100 bp (ThermoScientific, Baltics, UAB, Vilnius, Lithuania) was used to estimate the molecular weight of the PCR products. The electrophoretic profiles were then analyzed under UV light using the BioDoc-It Imaging System (Ultra-Violet Products Ltd., Upland, CA, USA).

### 4.3. In Vitro Evaluation of Antifungal Activity

The antifungal activity was tested against 15 pathogenic fungi (Table 8) using three different tests: a dual culture plate assay of bacterial endophytes, an agar diffusion assay of the CFSs (cell-free supernatants), and an inverted Petri dish assay for VOCs described by Silva-Campos et al. [110] and Yu et al. [111] with a few modifications.

#### 4.3.1. Antifungal Activity of Bacterial Endophytes

Bacterial endophytes were assessed in vitro using a dual culture assay on PDA medium. Briefly, bacterial strains were streaked at a 2 cm distance from the middle of the Petri plates (90 mm diameter). The fungi were placed in the center as fresh mycelial plugs of 6 mm diameter or as 6 µL spore suspension (10^4^ conidia/mL) for *Aspergillus* and *Penicillium* strains. For each fungal pathogen, pure cultures were prepared as control plates. The plates were then incubated at 25 °C in the dark. Clear inhibition zones were biometrically measured after 7 days of interaction. The antifungal efficacy (E%) was calculated as [112]

$$E\left(\%\right)=\frac{\mathrm{Rc}-\mathrm{Ri}}{\mathrm{Rc}}\times 100$$

where Ri—the fungal radius towards the biocontrol bacteria, Rc—the fungal radius in the control plates.

The antifungal effect of the tested bacterial strains was also observed via optical microscopy analysis. Direct microbial interactions were analyzed using the Nikon Eclipse 2000 LED MV R optical microscope (Nikon Corporation, Tokyo, Japan). Lactophenol cotton blue staining was applied to highlight the un-pigmented mycelia.

#### 4.3.2. Antifungal Activity of the CFSs

The CFSs from bacterial cultures were also tested for antifungal properties via the agar diffusion assay. Briefly, the supernatant was collected from 3-day-old liquid bacterial culture, centrifuged, and filtered through a sterile 0.22 µm Millipore membrane. For each antifungal test, PDA plates of 90 mm diameter were flooded with 800 µL CFS. After soaking, test plates were inoculated in the center with fresh mycelial plugs of 6 mm diameter or 6 µL spore suspension (10^4^ conidia/mL). Fungal control plates were also prepared. Incubation was carried out at 25 °C for 7 days. In order to carry out antagonism evaluation, the fungal growth was biometrically measured and the antifungal efficacy of the bacterial CFS was calculated.

#### 4.3.3. Antifungal Activity of the VOCs

The antifungal activity of the bacterial VOCs was also evaluated via the inverted Petri dish assay. For this test, 200 µL of fresh bacterial culture, with an OD of 0.3 at 600 nm, was spread on LB agar using sterile cotton inoculating swabs. Fungi were inoculated in the center of a PDA plate, as mycelial plugs of 6 mm diameter or as 6 µL spore suspension (10^4^ conidia/mL). These two types of inoculated plates, one with bacteria and the other with fungi, were placed together, and tightly sealed with parafilm foil. Control plates for each tested fungus were also prepared. The incubation was performed at 25 °C. The antifungal activity of VOCs was determined as in the previous test.

### 4.4. Enzymatic Activities of Bacterial Strains

Amylase, ACC-deaminase, arginine- and ornithine-decarboxylase, cellulase, chitinase, lipase, phytate, and protease activities were performed by inoculating the endophytic strains, in spots, on specific substrates.

1. Amylase activity was tested in nutrient agar supplemented with 0.4% soluble starch. The enzymatic activity was revealed after three days of incubation using Lugol’s solution. A clear halo around the bacterial growth was considered positive for amylase production.
2. ACC-deaminase activity was screened on a solid DF minimal medium [113], having 3 mM 1-aminocyclopropane-1-carboxylate (ACC) as a single nitrogen source. Tests were performed compared to positive and negative control plates. For the positive controls, the studied bacteria were inoculated on DF medium supplemented with 3 mM (NH_4_)_2_SO_4_ as the nitrogen source, while for the negative ones, DF minimal medium was used.
3. Arginine-decarboxylase as well as ornithine-decarboxylase activities were performed on solid media containing 0.2% L-arginine hydrochloride, or 0.2% L-ornithine hydrochloride, respectively, with phenol red as an indicator dye [114]. The enzymatic activity can be evaluated after one day of incubation due to the color change of the medium from yellow to pink.
4. Cellulase activity was screened on 1% carboxy-methyl cellulose (CMC)-supplemented medium [114] and developed using 0.3% Congo red staining for 15 min, followed by washing with NaCl 1 M for 15 min. A clear halo surrounding bacterial growth revealed positive results for cellulase production.
5. Xylanase activity was evaluated on xylan-containing medium for bacteria. The medium was prepared using 5 g/L peptone, 2 g/L yeast extract, 0.5 g/L magnesium sulfate, 0.5 g/L sodium chloride, 0.1 g/L calcium chloride, 5 g/L xylan, and 20 g/L agar. Positive strains were detected as in the previous test for cellulase-producing bacteria.
6. Chitinase activity was evaluated on Agrawal and Kotasthane medium based on colloidal chitin and bromocresol purple indicator dye [115]. Positive strains were detected through the color change of the substrate from yellow to purple.
7. The lipase test was carried out on a modified medium described by Sicuia et al. [114] containing calcium chloride (0.05%) and Tween 80 (1%) as inducers. The opacity areas surrounding the bacterial colonies indicated the presence of lipolytic activity.
8. Protease activity was evaluated by studying casein and gelatin hydrolyzation. The caseinolytic potential of the studied strains was evaluated on skim milk agar. The clear halo surrounding bacterial colonies was positively correlated to casein hydrolysis. Bacterial gelatin hydrolyzation potential was evaluated on gelatin solidified nutrient broth and compared to positive and negative controls. Substrate liquefaction was verified by submerging the culture tubes for 30 min in an ice bath. The samples that remained liquefied at a low temperature were gelatinase positive.
9. Phytate solubilization was performed on TS agar medium containing sodium phytate [116]. For this medium, 5 g/L glucose, 10 g/L bacto-peptone, 5 g/L yeast extract, 1 g/L magnesium sulfate, 1 g/L calcium chloride, and 2 g/L phytic acid sodium salt, final pH 7.0, were used. The bacterial strains that developed a clear halo around their colonies were phytase producers.
10. For API ZYM tests, the hydrolytic enzymes present in the grown cells were detected according to the API ZYM kit (BioMerieux, Marcy-l’Etoile, France) instructions for use. Briefly, freshly grown bacteria on TSA (Tryptic Soy Agar) were suspended in sterile saline solution 0.85%, until an approximately 0.75 absorbance was reached at 600 nm. After that, each cupule of the API ZYM strip was filled with 65 µL of cell suspension. The test strips were maintained at 30 °C in a plastic API strip incubation tray filled with 5 mL of sterile distilled water. After incubation, the reactions were developed by adding 1 drop of reagent A, followed by 1 drop of reagent B in each cupule. The reactions were let to develop under intensive light by placing the test strips for 5 to 10 min under 20,000 lx illuminance in a Sanyo MLR-351H chamber. When scoring the hydrolytic enzymes present in the grown cells, index values from 0 to 5 were attributed to each reaction. An index value of 0 corresponded to a negative reaction. The index 5 was given to maximum color intensity. Index values of 2 were scored as borderline (±), while index values from 3 to 5 were scored as positive (+).

### 4.5. Physiological Tests

Phosphate solubilization and surfactant production were performed by inoculating the endophytic strains, in spots, into specific substrates. Acetoin and ammonia production, as well as indole acetic acid synthesis, were highlighted in broth cultures under specific reaction conditions.

1. Phosphate solubilization was performed on NBRIP agar medium containing tricalcium phosphate [117]. For this medium, 10 g/L glucose, 0.1 g/L (NH_4_)_2_SO_4_, 0.2 g/L KCl, 5 g/L MgCl_2_·6H_2_O, 0.25 g/L MgSO_4_·7H_2_O, 5 g/L Ca_3_(PO_4_)_2_, and 15 g/L agar were used, at a final pH of 7.0. Positive strains were detected based on the clear halo surrounding the bacterial colonies.
2. Surfactant production on the CTAB medium was also tested. Bacteria were grown on a specific medium containing glucose 20 g/L, bacto-peptone 10 g/L, beef extract 1 g/L, yeast extract 0.5 g/L, CTAB 0.78 g/L, methylene blue 0.002 g/L, agar 17 g/L, and with a final pH 7.2. The positive strains were detected based on the clear halo surrounding the bacterial colonies.
3. Acetoin production was screened through the Voges–Proskauer test [118]. The test was performed on a liquid medium containing 7 g/L bacto-peptone, 5 g/L dextrose, and 5 g/L K_2_HPO_4_, with a pH of 7.0. The reaction was developed after 3 days of static incubation at 28 °C. For each 2 mL of bacterial culture, 6 drops of Barritt’s reagent A were used, followed by 2 drops of reagent B. Samples were homogenized after each step, then static incubated for 1h. Barritt’s reagent A contained 5% α-naphthol in absolute ethanol, while reagent B was an aqueous solution of 40% KOH.
4. The ammonia production test was performed in broth medium containing 5 g/L peptone, 3 g/L meat extract, and 2 g/L potassium nitrate, pH 7.0. The reaction was developed with Nessler’s reagent, in a 9:1 (*v*/*v*) proportion, after 4 days of static incubation at 28 °C. Positive ammonia producers formed a brown-yellow precipitate.
5. Indole-3-acetic acid (IAA) was quantified in LB broth and LB supplemented with 2.5 mM tryptophan as the IAA precursor. IAA production was quantified spectrophotometrically after 1 to 3 days of incubation at 28 °C, with 120 rpm shaking. For each 1 mL of centrifuged culture supernatant, 2 drops of o-phosphoric acid were added, then 2 mL of Salkowski reagent (FeCl_3_-HClO_4_). After 30 min of incubation, samples were read at 530 nm. A standard curve containing 0 ÷ 30 mg IAA/mL was also prepared to perform IAA quantification.

### 4.6. Ecological Tests

The endophytes’ potential to grow at different temperatures, pH values, and CuSO_4_ concentrations was evaluated by inoculating an LB-agar-based medium with 10 µL of bacterial culture with an OD of 0.3 at 600 nm.

1. The pH resistance test was performed to detect the growth potential of the studied endophytic bacteria at pH values of 5, 7, 8, 9, 10, and 11 ± 0.2. The study was performed on LB agar where the pH was modified with 0.2 N HCl or 1 N NaOH until the desired pH value was reached. Incubation was carried out at 30 °C, and plates were analyzed daily.
2. The temperature tolerance test was performed to detect the growth potential of the studied endophytic bacteria at 10, 20, 30, 40, and 50 °C. The study was performed on LB agar at a pH 7.0.
3. The CuSO_4_ tolerance test was performed on an LB medium supplemented with 50 to 700 mg/L of CuSO_4_.5H_2_O, according to Vörös et al. [100], with few modifications. Incubation was carried out at 30 °C, and plates were analyzed until the fifth day.

### 4.7. Statistical Analysis

Experiments were performed in triplicate and the results were represented as mean ± standard deviation. Significant differences between the antifungal activity methods used, in terms of antifungal efficacy, were assessed using analysis of variance (ANOVA) with the Tukey B post hoc test (*p* < 0.05). Groups with different letters are considered significantly different at a *p*-value less than 0.05. Statistical analysis was performed using SPSS version 28.0 (IBM Corp., Armonk, NY, USA) software.

## 5. Conclusions

Based on the 16S rDNA sequence analysis, six bacterial endophytic strains isolated from different plant organs and species were identified as belonging to *Bacillus* spp., such as *B. velezensis*, *B. pumilus*, and *B. subtilis*. The strong antifungal activity of *B. velezensis* strains, against a wide range of pathogenic fungi associated with GTDs and grape spoilage, could be correlated with the molecular detection of different genes involved in the biosynthesis of various lipopeptides (bacillomycin, iturins, fengycin, and surfactin) as well as volatile compounds, enzymes, and other bioactive metabolites. Among all tested strains, BAHs1 seems to be the most effective in inhibiting fungal growth. Different bacterial strains were found to have the ability to synthesize ACC deaminase, IAA, surfactant, acetoin, ammonia, as well as aid in phosphate solubilization. The survival of *Bacillus* strains under adverse environmental conditions (extreme temperature and pH) could be correlated with their ability to form endospores, which makes them ideal to be used as biocontrol agents. In future, the *B. velezensis* BAHs1 strain will be validated, *in vivo*, as a biocontrol agent to evaluate its ability to protect grapevine and grapes against fungal pathogens in both pot and post-harvest experiments. Moreover, future research aims to study the possible synergic effects for a complex consortia of bacterial strains, combined with *Trichoderma* spp., to develop appropriate formulations and application techniques to remove or reduce the fungal incidence in vineyards.

## Figures and Tables

**Table 1:** Antifungal activity of plant beneficial endophytes.

Fungal Species	*In Vitro* Fungal Growth Inhibition Efficacy of Plant Beneficial Endophytes					
	LFF MYM 5	St 1T2	E1Pv	BAHs1	BPVs2	BTAs3
*Spoilage fungi*						
*Aspergillus carbonarius* MI 15	80.6 ± 3.1 ^a^	53.5 ± 27.8 ^c^	74.9 ± 1.0 ^b^	81.1 ± 1.2 ^a^	75.9 ± 1.6 ^b^	78.1 ± 2.8 ^ab^
*A. flavus* MI 24	58.6 ± 12.4 ^b^	0.0 ^e^	6.4 ± 3.3 ^d^	68.5 ± 1.6 ^a^	40.0 ± 3.8 ^c^	62.9 ± 1.9 ^b^
*A. niger* MI 5	78.7 ± 3.6 ^a^	4.1 ± 2.8 ^e^	45.6 ± 3.8 ^d^	77.3 ± 2.9 ^a^	66.4 ± 3.4 ^c^	69.2 ± 3.1 ^bc^
*A. ochraceus* MI 2	82.2 ± 0.5 ^a^	4.5 ± 5.1 ^e^	68.4 ± 0.6 ^d^	80.2 ± 5.1 ^ab^	75.3 ± 1.3 ^c^	78.7 ± 1.2 ^b^
*Botrytis cinerea* MI Aligote Husi	73.4 ± 5.8 ^a^	62.9 ± 0.5 ^a^	75.3 ± 9.9 ^a^	77.7 ± 11.7 ^a^	76.3 ± 12.0 ^a^	77.9 ± 11.4 ^a^
*P. expansum* MI BB Husi	70.5 ± 2.1 ^ab^	35.9 ± 22.4 ^c^	42.7 ± 1.9 ^c^	72.5 ± 5.0 ^a^	54.5 ± 20.5 ^b^	66.5 ± 3.5 ^b^
*Trunk disease fungi*						
*C. rosea* MI 1	55.0 ± 2.5 ^c^	3.3 ± 2.9 ^d^	55.0 ± 2.9 ^c^	67.5 ± 2.0 ^a^	60.0 ± 2.5 ^b^	65.0 ± 2.5 ^ab^
*D. seriata* CBS 151.24	69.3 ± 9.3 ^a^	31.6 ± 14.2 ^c^	49.0 ± 5.6 ^bc^	75.7 ± 13.6 ^a^	66.4 ± 15.5 ^ab^	74.4 ± 12.6 ^a^
*E. lata* CBS 208.87	81.1 ± 3.8 ^a^	48.6 ± 16.1 ^b^	75.1 ± 3.9 ^a^	82.3 ± 8.1 ^a^	78.0 ± 7.4 ^a^	82.2 ± 7.4 ^a^
*Fusarium equiseti* MI 6	58.8 ± 1.2 ^b^	10.5 ± 5.1 ^c^	58.1 ± 1.3 ^b^	61.0 ± 2.4 ^ab^	58.8 ± 3.5 ^b^	58.5 ± 1.0 ^b^
*F. oxysporum* MI 3	73.0 ± 4.6 ^a^	5.1 ± 3.9 ^e^	45.6 ± 1.3 ^d^	71.6 ± 0.7 ^a^	57.0 ± 5.0 ^c^	63.1 ± 3.8 ^b^
*F. proliferatum* MI 4	76.3 ± 2.1 ^a^	8.6 ± 4.5 ^e^	64.1 ± 1.3 ^cd^	72.4 ± 1.9 ^b^	61.8 ± 2.6 ^d^	69.7 ± 4.6 ^bc^
*F. solani* MI 13	64.0 ± 1.5 ^b^	7.9 ± 2.6 ^d^	61.0 ± 0.8 ^c^	64.5 ± 1.5 ^b^	61.4 ± 1.5 ^c^	68.0 ± 0.8 ^a^
*N. parvum* MI 25	65.8 ± 1.4 ^b^	0.0 ^d^	36.7 ± 11.5 ^c^	79.0 ± 6.4 ^a^	63.3 ± 1.4 ^b^	64.2 ± 2.9 ^b^
*S. hirsutum* CBS416.61	87.4 ± 1.2 ^a^	23.2 ± 13.3 ^c^	66.3 ± 6.3 ^b^	86.1 ± 6.1 ^a^	80.5 ± 2.4 ^a^	85.8± 4.4 ^a^

The data are presented as average values ± standard deviation. Different letters attributed to the same fungal interaction indicate a significant difference between the experimental variants, regarding their fungal inhibitory activity.

**Table 2:** Antifungal activity of bacterial CFSs against grapevine pathogens.

Fungal Species	*In Vitro* Fungal Growth Inhibition Efficacy of Plant Beneficial Endophytes CFSs					
	LFF MYM 5	St 1T2	E1Pv	BAHs1	BPVs2	BTAs3
***Spoilage fungi***						
*Aspergillus carbonarius* MI 15	15.8 ± 1.8 ^b^	2.6 ± 1.3 ^d^	7.5 ± 2.0 ^c^	18.0 ± 2.8 ^b^	23.5 ± 0.9 ^a^	18.7 ± 2.3 ^b^
*A. flavus* MI 24	21.3 ± 6.7 ^ab^	1.8 ± 0.8 ^d^	6.9 ± 3.0 ^c^	17.5 ± 6.5 ^b^	12.7 ± 4.6 ^b^	16.6 ± 5.2 ^b^
*A. niger* MI 5	14.7 ± 5.1 ^a^	2.8 ± 2.3 ^b^	8.6 ± 7.1 ^ab^	14.0 ± 8.1 ^ab^	18.4 ± 7.6 ^a^	8.8 ± 4.5 ^ab^
*A. ochraceus* MI 2	34.3 ± 6.2 ^a^	5.9 ± 5.5 ^b^	11.3 ± 10.6 ^b^	36.3 ± 6.4 ^a^	31.5 ± 6.0 ^a^	32.5 ± 5.7 ^a^
*Botrytis cinerea* MI Aligote Husi	22.3 ± 2.7 ^a^	1.9 ± 1.9 ^c^	3.7 ± 3.5 ^c^	31.4 ± 6.0 ^a^	18.1 ± 1.9 ^b^	28.4 ± 6.5 ^a^
*P. expansum* MI BB Husi	51.2 ± 3.7 ^a^	43.6 ± 0.3 ^c^	46.4 ± 2.5 ^bc^	47.7 ± 3.1 ^bc^	45.8 ± 3.6 ^bc^	51.2 ± 2.7 ^a^
***Trunk disease fungi***						
*C. rosea* MI 1	61.9 ± 2.6 ^c^	54.7 ± 8.1 ^c^	60.1 ± 3.8 ^bc^	65.8 ± 2.3 ^a^	63.4 ± 2.3 ^ab^	62.4 ± 1.6 ^bc^
*D. seriata* CBS 151.24	64.6 ± 3.5 ^b^	27.8 ± 1.9 ^e^	44.5 ± 7.1 ^d^	63.4 ± 4.8 ^b^	56.6 ± 6.0 ^c^	71.5 ± 3.9 ^a^
*E. lata* CBS 208.87	39.5 ± 5.8 ^a^	19.4 ± 6.3 ^b^	41.0 ± 7.8 ^a^	49.3 ± 5.5 ^a^	45.2 ± 3.9 ^a^	45.5 ± 3.3 ^a^
*Fusarium equiseti* MI 6	4.9 ± 2.8 ^a^	0.0 ^b^	1.9 ± 1.3 ^ab^	4.0 ± 4.0 ^ab^	7.2 ± 2.4 ^a^	5.6 ± 2.6 ^a^
*F. oxysporum* MI 3	34.3 ± 4.3 ^a^	4.5 ± 3.9 ^b^	9.4 ± 4.5 ^b^	41.6 ± 3.7 ^a^	36.1 ± 5.1 ^a^	34.9 ± 6.1 ^a^
*F. proliferatum* MI 4	30.0 ± 1.9 ^a^	7.8 ± 7.1 ^b^	17.6 ± 8.5 ^b^	35.0 ± 1.5 ^a^	32.3 ± 2.3 ^a^	32.5 ± 2.4 ^a^
*F. solani* MI 13	24.6 ± 2.2 ^a^	2.3 ± 2.2 ^c^	6.6 ± 1.2 ^b^	21.0 ± 3.5 ^a^	21.3 ± 2.1 ^a^	20.7 ± 2.1 ^a^
*N. parvum* MI 25	68.5 ± 1.7 ^a^	46.4 ± 4.9 ^c^	59.8 ± 2.7 ^b^	69.7 ± 4.1 ^a^	68.5 ± 3.1 ^a^	70.5 ± 3.3 ^a^
*S. hirsutum* CBS416.61	50.9 ± 6.2 ^a^	12.6 ± 2.5 ^c^	37.7 ± 3.1 ^b^	55.7 ± 4.6 ^a^	54.8 ± 4.6 ^a^	55.4 ± 6.5 ^a^

The data are presented as average values ± standard deviation. Different letters attributed to the same fungal interaction indicate a significant difference between the experimental variants regarding their fungal inhibitory activity.

**Table 3:** Antifungal activity of bacterial VOCs against grapevine pathogens.

Fungal Species	*In Vitro* Fungal Growth Inhibition Efficacy of Plant Beneficial Endophytes VOCs					
	LFF MYM 5	St 1T2	E1Pv	BAHs1	BPVs2	BTAs3
***Spoilage fungi***						
*Aspergillus carbonarius* MI 15	69.7 ± 6.7 ^a^	62.6 ± 3.6 ^a^	65.1 ± 0.1 ^a^	76.3 ± 7.2 ^a^	67.4 ± 3.4 ^a^	69.2 ± 4.1 ^a^
*A. flavus* MI 24	43.4 ± 1.1 ^b^	44.1 ± 0.1 ^b^	27.8 ± 3.5 ^c^	42.8 ± 0.3 ^b^	47.2 ± 0.7 ^a^	49.1 ± 0.4 ^a^
*A. niger* MI 5	10.5 ± 4.2 ^ab^	8.1 ± 1.2 ^b^	7.4 ± 1.8 ^b^	18.2 ± 4.1 ^a^	11.6 ± 2.3 ^ab^	12.4 ± 3.7 ^ab^
*A. ochraceus* MI 2	72.1 ± 6.2 ^a^	46.3 ± 5.7 ^b^	50.8 ± 5.2 ^b^	73.8 ± 5.0 ^a^	62.9 ± 3.1 ^a^	67.1 ± 4.4 ^a^
*Botrytis cinerea* MI Aligote Husi	77.9 ± 1.6 ^ab^	43.0 ± 1.6 ^c^	67.4 ± 3.3 ^b^	84.9 ± 1.6 ^a^	81.4 ± 3.3 ^a^	83.7 ± 3.3 ^a^
*P. expansum* MI BB Husi	69.0 ± 1.4 ^a^	54.3 ± 3.3 ^b^	53.7 ± 0.5 ^b^	72.5 ± 3.5 ^a^	73.0 ± 4.2 ^a^	69.3 ± 10.4 ^a^
***Trunk disease fungi***						
*C. rosea* MI 1	47.1 ± 3.1 ^abc^	39.2 ± 5.2 ^c^	40.0 ± 4.3 ^bc^	55.8 ± 5.1 ^a^	49.2 ± 3.8 ^ab^	50.8 ± 3.8 ^a^
*D. seriata* CBS 151.24	74.8 ± 3.0 ^b^	55.0 ± 3.1 ^c^	54.5 ± 4.3 ^c^	83.7 ± 0.7 ^a^	72.8 ± 3.8 ^b^	73.8 ± 2.2 ^b^
*E. lata* CBS 208.87	68.8 ± 0.4 ^b^	61.5 ± 0.6 ^c^	59.4 ± 0.2 ^d^	73.3 ± 4.1 ^b^	63.6 ± 0.9 ^bc^	78.2 ± 0.6 ^a^
*Fusarium equiseti* MI 6	1.6 ± 1.2 ^a^	1.6 ± 1.2 ^a^	1.6 ± 1.2 ^a^	1.7 ± 0.8 ^a^	1.6 ± 1.2 ^a^	1.6 ± 1.2 ^a^
*F. oxysporum* MI 3	17.8 ± 1.3 ^a^	16.3 ± 4.0 ^a^	17.1 ± 1.3 ^a^	20.5 ± 4.1 ^a^	19.4 ± 1.3 ^a^	18.6 ± 2.3 ^a^
*F. proliferatum* MI 4	41.9 ± 2.3 ^ab^	37.2 ± 3.1 ^bc^	38.0 ± 3.6 ^abc^	43.8 ± 2.4 ^a^	41.9 ± 2.3 ^ab^	32.9 ± 2.9 ^c^
*F. solani* MI 13	3.1 ± 2.6 ^a^	2.7 ± 1.9 ^a^	5.3 ± 5.0 ^a^	6.4 ± 3.8 ^a^	5.0 ± 3.5 ^a^	6.3 ± 3.4 ^a^
*N. parvum* MI 25	72.6 ± 5.1 ^a^	65.5 ± 5.1 ^a^	67.9 ± 5.1 ^a^	77.4 ± 1.7 ^a^	77.4 ± 1.7 ^a^	76.8 ± 0.8 ^a^
*S. hirsutum* CBS 416.61	79.3 ± 2.3 ^b^	75.3 ± 2.1 ^c^	74.0 ± 3.0 ^c^	85.8 ± 3.6 ^a^	79.7 ± 0.5 ^b^	82.5 ± 1.2 ^a^

The data are presented as average values ± standard deviation. Different letters attributed to the same fungal interaction indicate a significant difference between the experimental variants regarding their fungal inhibitory activity.

**Table 4:** Extracellular enzymatic activities of bacterial strains.

Bacterial Strains	Amy	ACC-Deam	Arg	Orn	Cel	Xyl	Chi	Lip	Cas	Gel	Phyt
LFF MYM 5	+	++	++	+	+	−	−	−	+	+	+
St 1T2	−	+	±	++	++	−	−	+	+++	+	−
E1Pv	±	++	++	+	+++	−	+	++	++	+	−
BAHs1	+	+	++	±	++	−	−	−	+	+	+
BPVs2	+	−	+	±	+	−	−	−	+	+	+
BTAs3	+	+	+	±	+	−	−	−	+	+	+

Legend: Amy = α-amylase, ACC-deam = ACC-deaminase, Arg = arginine-decarboxylase, Orn = ornithine-decarboxylase, Cel = cellulase activity, Xyl = xylanase activity, Chi = chitinase activity, Lip = Tween 80 lipase, Cas = caseinase, Gel = gelatinase, Phyt = phytase activity. Note: − negative reaction, ± borderline, + positive reaction. In semiquantitative estimation, multiple + symbols mean an increased positive reaction.

**Table 5:** Enzymatic activity of the bacterial strains based on API ZYM system.

Bacterial Strains	Hydrolytic Enzymes in Grown Cells	Reaction
LFF MYM 5	Alkaline phosphatase	±
	Esterase	±
	Esterase Lipase	±
	Naphthol-AS-BI-phosphohydrolase	+
St 1T2	Esterase	+
	Esterase Lipase	+
	α-chymotrypsin	+
E1Pv	Alkaline phosphatase	±
	Esterase	+
	Esterase Lipase	+
	α-glucosidase	+
	β-glucosidase	+
BAHs1	Alkaline phosphatase	±
	Esterase	±
	Esterase Lipase	+
	Acid phosphatase	+
	Naphthol-AS-BI-phosphohydrolase	±
BPVs2	Alkaline phosphatase,	+
	Esterase,	+
	Esterase Lipase	+
	Acid phosphatase	+
BTAs3	Alkaline phosphatase	+
	Esterase	±
	Esterase Lipase	+
	Acid phosphatase	±

± Borderline, + positive reaction.

**Table 6:** Biochemical characterization of bacterial strains.

Bacterial Strains	Phos	VP	NH_3_	CTAB	IAA (µg/mL) on LB			IAA (µg/mL) on LB with 2.5 mM Tryptophan		
					24 h	48 h	72 h	24 h	48 h	72 h
LFF MYM 5	−	++	++	−	1.85 ± 0.3 ^a^	3.34 ± 0.2 ^b^	5.01 ± 0.3 ^a^	1.20 ± 0.3 ^a^	3.24 ± 0.4 ^abc^	7.03 ± 1.1 ^a^
St 1T2	++	++	+	−	0.30 ± 0.2 ^d^	0.68 ± 0.2 ^d^	0.80 ± 0.3 ^c^	1.08 ± 0.4 ^a^	1.65 ± 0.6 ^d^	3.98 ± 1.1 ^b^
E1Pv	+	+++	++	−	0.80 ± 0.2 ^bc^	2.18 ± 0.3 ^c^	3.83 ± 0.3 ^b^	1.08 ± 0.3 ^a^	4.18 ± 0.7 _ab_	8.62 ± 1.1 ^a^
BAHs1	−	+	++	+	0.68 ± 0.1 ^bcd^	1.10 ± 0.3 ^d^	1.15 ± 0.2 ^c^	0.75 ± 0.3 ^a^	3.06 ± 0.4 ^bc^	4.16 ± 0.3 ^b^
BPVs2	−	−	++	−	0.53 ± 0.1 ^bcd^	4.17 ± 0.2 ^a^	5.00 ± 0.2 ^a^	0.83 ± 0.3 ^a^	3.38 ± 0.1 ^cd^	2.46 ± 0.9 ^b^
BTAs3	−	++++	++	+	0.50 ± 0.2 ^cd^	4.07 ± 0.2 ^a^	4.69 ± 0.4 ^a^	1.93 ± 0.3 ^a^	4.07 ± 0.8 ^a^	6.14 ± 1.1 ^a^

Legend: Phos = phosphate solubilization, VP = Voges–Proskauer test for acetoin production, NH_3_ = ammonia production, CTAB = surfactant production. Note: + positive reaction, ± borderline, − negative reaction. In semiquantitative estimation, multiple + symbols mean an increased positive reaction. Different letters denote that mean values are significantly different, at a significance level of *p* ≤ 0.05.

**Table 7:** Effect of several abiotic factors on the bacterial growth.

Bacterial Strains	pH Tolerance						Growth Temperature (°C)					Cooper Tolerance (mg CuSO_4_/L)			
	5	7	8	9	10	11	10	20	30	40	50	0	50	100	200 ÷ 700
LFF MYM 5	+	+	+	+	+	+	−	+	+	+	+	+	−	−	−
St 1T2	+	+	+	+	+	+	+	+	+	+	+	+	+	+	+
E1Pv	+	+	+	+	+	+	−	+	+	+	+	+	+	+	−
BAHs1	+	+	+	+	+	+	−	+	+	+	+	+	−	−	−
BPVs2	+	+	+	+	+	+	−	+	+	+	+	+	±	−	−
BTAs3	+	+	+	+	+	+	−	+	+	+	+	+	−	−	−

± Borderline, + positive reaction, − negative reaction.

**Table 8:** Fungal pathogens and reference numbers.

Species Name	Strain	Isolation Source	Microbial Collection
*Aspergillus carbonarius*	MI 15	Table grapes	UASVM Bucharest
*A. flavus*	MI 24	Grapes	UASVM Bucharest
*A. niger*	MI 5	Table grapes	UASVM Bucharest
*A. ochraceus*	MI 2	Dried grapes	UASVM Bucharest
*Botrytis cinerea*	MI Aligote Huși	Aligote grapes	UASVM Bucharest
*Clonostachys rosea*	MI 1	-	UASVM Bucharest
*Diplodia seriata*	CBS 151.24	-	CBS, Westerdijk Institute, The Netherlands
*Eutypa lata*	CBS 208.87	Host plant -*Tilia* sp. Origin–Switzerland	CBS, Westerdijk Institute, The Netherlands
*Fusarium equiseti*	MI 6	Natural contaminant of esca diseased grapevine	UASVM Bucharest
*F. oxysporum*	MI 3	Natural contaminant of esca diseased grapevine	UASVM Bucharest
*F. proliferatum*	MI 4	Natural contaminant of esca diseased grapevine	UASVM Bucharest
*F. solani*	MI 13	Natural contaminant of esca diseased grapevine	UASVM Bucharest
*Neofusicoccum parvum*	MI 25	Natural contaminant of esca diseased grapevine	UASVM Bucharest
*Penicillium expansum*	MI BB Huși	Grapes from Huși vineyard	UASVM Bucharest
*Stereum hirsutum*	CBS 416.61	-	CBS, Westerdijk Institute, The Netherlands

**Table 9:** Primers used in this study.

Antifungal Compound	Gene	Primers	Primer Sequence 5′–3′	PCR Product (bp)	Annealing Temperature	References
bacillibectin	*BAC*	Bac-F	ATCTTTATGGCGGCAGTC	595	58 °C	[105]
		Bac-R	ATACGGCTTACAGGCGAG			
difficidin	*dfnA*	dfnA F	GGATTCAGGAGGGCATACCG	653	55 °C	[106]
		dfnA R	ATTGATTAAACGCGCCGAGC			
fengycin	*Fen*	Af2 F	GAATAYMTCGGMCGTMTKGA	443, 452	45 °C	[107]
		Tf1 R	GCTTTWADKGAATSBCCGCC			
iturin C	*itu C*	ITUC F1	CCCCCTCGGTCAAGTGAATA	594	55 °C	[108]
		ITUC R1	TTGGTTAAGCCCTGATGCTC			
iturin D	*ituD*	ITUD F1	TTGAAYGTCAGYGCSCCTTT	482	55 °C	[108]
		ITUD R1	TGCGMAAATAATGGSGTCGT			
iturin A synthetase D	*ituAD*	ituD-F	CCCCTGTTCTAGATGATCGGAGGAATCTC	800	55 °C	[109]
		ituD-R	TGCATCGATTCTGTCCATCTAACCGGCATC			
macrolactin	*mnlA*	mlnA F	CCGTGATCGGACTGGATGAG	668	55 °C	[106]
		mlnA R	CATCGCACCTGCCAAATACG			
mycosubtilin	*myc*	Am1 F	CAKCARGTSAAAATYCGMGG	416, 419	45 °C	[107]
		Tm1 R	CCDASATCAAARAADTTATC			

## Data Availability

Not applicable.
